# Post-Injury Neuroprotective Effects of the Thalidomide Analog 3,6′-Dithiothalidomide on Traumatic Brain Injury

**DOI:** 10.3390/ijms20030502

**Published:** 2019-01-24

**Authors:** Buyandelger Batsaikhan, Jing-Ya Wang, Michael T. Scerba, David Tweedie, Nigel H. Greig, Jonathan P. Miller, Barry J. Hoffer, Chih-Tung Lin, Jia-Yi Wang

**Affiliations:** 1 Graduate Institute of Medical Sciences, College of Medicine, Taipei Medical University, 250 Wu-Xing Street, Taipei 11031, Taiwan; buyndlgr@gmail.com (B.B.); gaeajyw911real@hotmail.com.tw (J.-Y.W.); sss880205@gmail.com (C.-T.L.); 2 Drug Design & Development Section, Translational Gerontology Branch, Intramural Research Program, National Institute on Aging, NIH, Baltimore, MD 21224, USA; mike.scerba@nih.gov (M.T.S.); tweedieda@grc.nia.nih.gov (D.T.); greign@grc.nia.nih.gov (N.H.G.); 3 Department of Neurological Surgery, Case Western Reserve University, Cleveland, OH 44106, USA; Jonathan.Miller@uhhospitals.org (J.P.M.); bjh82@case.edu (B.J.H.); 4 Department of Physiology, School of Medicine, College of Medicine, Taipei Medical University, Taipei 11031, Taiwan

**Keywords:** traumatic brain injury, neurological deficits, 3,6′-dithiothalidomide, neurodegeneration, neuroinflammation, oxidative stress

## Abstract

Traumatic brain injury (TBI) is a major cause of mortality and disability worldwide. Long-term deficits after TBI arise not only from the direct effects of the injury but also from ongoing processes such as neuronal excitotoxicity, inflammation, oxidative stress and apoptosis. Tumor necrosis factor-α (TNF-α) is known to contribute to these processes. We have previously shown that 3,6′-dithiothalidomide (3,6′-DT), a thalidomide analog that is more potent than thalidomide with similar brain penetration, selectively inhibits the synthesis of TNF-α in cultured cells and reverses behavioral impairments induced by mild TBI in mice. In the present study, we further explored the therapeutic potential of 3,6′-DT in an animal model of moderate TBI using Sprague-Dawley rats subjected to controlled cortical impact. A single dose of 3,6′-DT (28 mg/kg, i.p.) at 5 h after TBI significantly reduced contusion volume, neuronal degeneration, neuronal apoptosis and neurological deficits at 24 h post-injury. Expression of pro-inflammatory cytokines in the contusion regions were also suppressed at the transcription and translation level by 3,6′-DT. Notably, neuronal oxidative stress was also suppressed by 3,6′-DT. We conclude that 3,6′-DT may represent a potential therapy to ameliorate TBI-induced functional deficits.

## 1. Introduction

Traumatic brain injury (TBI), one of the leading causes of disability, morbidity, and mortality worldwide, currently has no effective treatment. The deficits associated with TBI arise both from direct mechanical effects (primary injury) and indirect effects arising from complex pathological cascades involving a broad spectrum of cellular and molecular pathways (secondary injury). The primary brain injury occurs at the time of initial impact and produces a series of direct insults such as acute cell death due to mechanical disruption. Secondary injury arises from subsequent physiological processes such as excitotoxicity that triggers a number of events including peri-lesion depolarization and the more delayed mechanisms of inflammation and apoptosis. The inflammatory response from activated microglia, recruited neutrophils and macrophages, and oxidative stress, contribute to secondary brain damage and, subsequently, lead to a progressive pathology [1,2,3,4] producing additional injury to neural tissues [5]. Therapeutic options to treat primary injury are limited since these processes are usually complete by the time of clinical presentation, but modulation of secondary injury pathways in the early-injury period has considerable clinical potential to improve outcomes after TBI.

Tumor necrosis factor-α (TNF-α) is a multifunctional pro-inflammatory cytokine involved in neuroinflammatory cascades associated with CNS injury. TNF-α is known to play an important role in initiating and propagating the inflammatory response after TBI [6]. In post-mortem brain samples from patients who died shortly after TBI, TNF-α mRNA and protein levels become acutely elevated within as little as 17 min after injury [7]. Likewise, in rodent TBI animal models, an acute rise in TNF-α precedes the rise of other pro-inflammatory cytokines [8,9,10]. TNF-α can exacerbate trauma and oxidative stress within the brain and contribute to glutamate release and blood-brain barrier dysfunction that leads to further influx of inflammatory factors from blood to brain [11,12]. There has been considerable interest in the development of drugs that block the pathological actions of TNF-α in a range of inflammatory conditions. TNF-α antibodies have been shown to be effective for the treatment of arthritis, inflammatory bowel disorders, and sepsis [13,14,15]; however, antibody therapy is not effective for brain trauma because circulating antibodies have limited access to the brain following routine systemic administration. Another option is to use small molecules that cross the blood-brain barrier for pharmacological manipulation of these processes.

**Figure 1:**
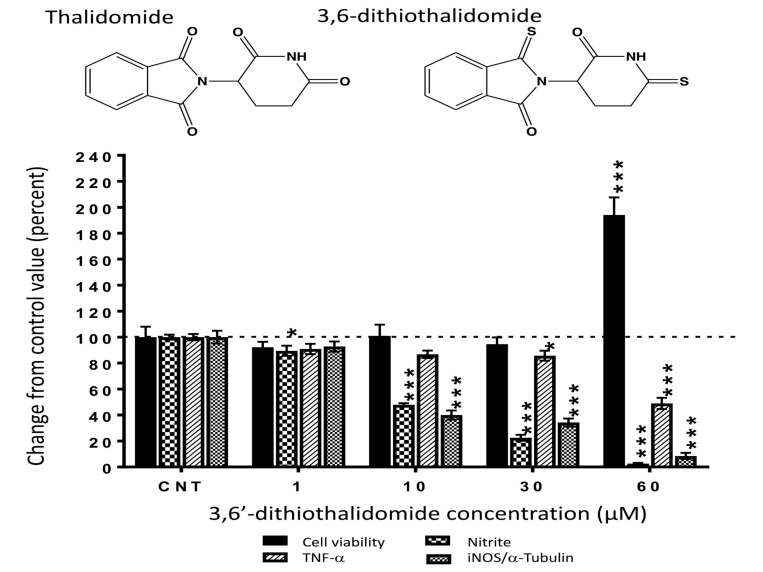
3,6′-dithiothalidomide (3,6′-DT) significantly attenuates the generation of TNF-α and nitrite, and the induction of iNOS protein in LPS activated RAW 264.7 cells. Media nitrite levels were significantly reduced at all 3,6′-DT concentrations evaluated. Media TNF-α protein levels were significantly reduced at 30 µM 3,6′-DT. Cellular levels of iNOS were significantly decreased in drug treated cells at a concentration of 10 and 30 µM. Only cells treated with 3,6′-DT at 60 µM indicated any evidence of cell toxicity, as determined by elevations in the absorbance of culture media observed from the lactate dehydrogenase assay (LDH). Drug effects on TNF-α, nitrite and iNOS at concentrations of 1 to 30 µM, were due to selective anti-inflammatory actions of the drug. Values are expressed as a percent change from drug vehicle control levels, the data are presented as mean ± S.E.M (*n* = 3 to 4 culture wells per group). * *p* < 0.05 and *** *p* < 0.001 compared with the respective control (CNT) group to which vehicle alone was added.

Thalidomide (Figure 1) is a small molecule that profoundly and reversibly inhibits TNF-α production, and is able to pass blood-brain barrier. It was initially introduced as a sedative hypnotic but was withdrawn from the market because of its teratogenic effects [16], and it has recently been reintroduced for the treatment of erythema nodosum leprosum, and cancer [17]. This clinical efficacy is based on its ability to inhibit the transcription of *TNF-α* [18] and *IL-6* [19], as well as repress TNF-α translation [20]. Recent preclinical studies suggest that thalidomide might also be effective for neurological damage since it protects neurons and improves functional outcome in animal models excitotoxic injury to striatal neurons [21].

In a recent clinical trial for Alzheimer’s disease, these adverse effects limited the thalidomide dose to a subtherapeutic and non-efficacious levels [22]. In order to further develop this therapeutic strategy, it is important to investigate the mechanisms of this drug and to develop moieties that enhance efficacy while minimizing side effects. To accomplish this, we previously developed and tested a series of thiothalidomide analogs and found that 3,6′-dithiothalidomide (3,6′-DT, Figure 1) was particularly effective [23,24,25]. Our previous investigations have shown that both thalidomide and 3,6′-DT can inhibit TNF-α production in cultured immune cells [25]. Moreover, as assessed by its brain/plasma ratio of 1.34, 3,6′-DT readily enters the CNS and reduces TNF-α levels in the brain in animal models of neuroinflammation [26,27,28]. We have also shown that 3,6′-DT reduces neuronal loss and improves functional outcome in a mouse model of focal ischemic stroke [29] and mild TBI [30]. In the present study, we further explored the therapeutic potential and mechanisms of 3,6′-DT in a rat model of moderate TBI using controlled cortical impact (CCI).

## 2. Results

### 2.1. 3,6′-DT Lowers Markers of Inflammation in Cellular Studies

The treatment of cultured RAW 264.7 cells with 3,6′-DT significantly reduced the levels of LPS-induced TNF-α and nitrite measured in the cell culture media in the absence of cell toxicity. Notably, the actions of 3,6′-DT on nitrite generation were more pronounced when compared to the effects on TNF-α generation. Nitrite (NO_2_^−^) is a major oxidation product derived from nitrogen monoxide (NO) that is produced within a wide variety of cell types by nitric oxide synthases. Media nitrite levels were significantly reduced, compared to TBI + Veh controls, at 3,6′-DT concentrations as low as 1 µM. While TNF-α levels were lower in drug-treated cell culture media, the reductions only became significant at a concentration of 30 µM or more. The effects of 3,6′-DT on protein levels of inducible nitric oxide synthase (iNOS) were evaluated and were similarly regulated by the drug (Figure 1). In contrast, no actions of thalidomide were measured on these parameters [31]. Due to the presence of cell toxicity at 60 µM, we cannot discriminate between selective drug effects on any of the measured variables; as such, we do not discuss the effects of the drug at 60 µM on cellular iNOS/media NO_2_^−^ or TNF-α. The observed changes on the variables may be due to cell death and not selective anti-inflammatory activity at this concentration.

### 2.2. Post-Injury 3,6′-DT Treatment Significantly Reduced Contusion Volume

**Figure 2:**
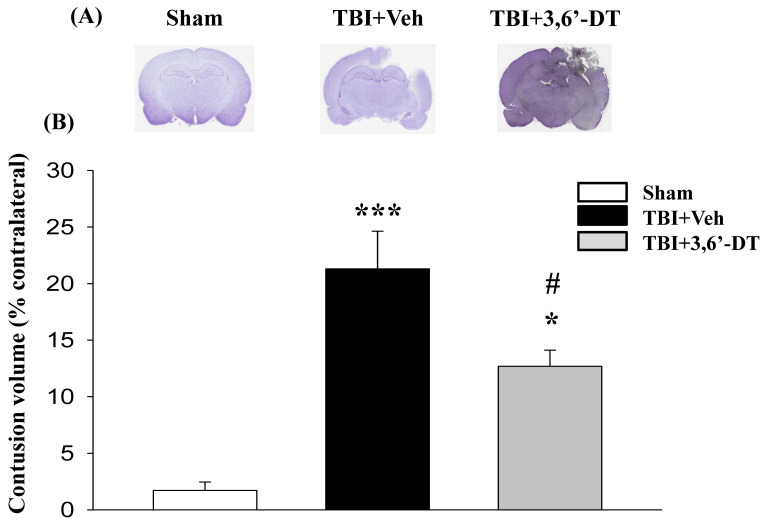
Post-injury administration of 3,6′-DT at 5 h after Traumatic brain injury (TBI) significantly reduced contusion volume evaluated at 24 h. (**A**) Representative cresyl violet stained coronal brain sections of TBI-induced cavity in Sham, TBI-Vehicle (TBI + Veh), and 3,6′-DT-treated TBI rats (TBI + 3,6′-DT) at 24 h post-TBI. (**B**) The TBI-induced contusion volume evaluated at 24 h was significantly reduced by 3,6′-DT treatment. Data are presented as mean ± S.E.M. (*n* = 5 in each group). * *p* < 0.05 and *** *p* < 0.001 compared with the Sham group. ^#^ *p* < 0.01 compared with the TBI + Veh group.

We measured the ipsilateral hemisphere for contusion volume (as a % of contralateral) for different treatments at 24 h after CCI. As in previous studies, CCI injury caused neuronal cell death resulting in a loss of cortical tissue in the ipsilateral hemisphere as a result of contusion volume (Figure 2A). Post-injury administration of 3,6′-DT (28 mg/kg) at 5 h significantly reduced contusion volume to 12.69 ± 1.42% (Figure 2B) as compared to TBI + Veh animals (21.2 ± 3.0% volume) (*p* < 0.01); representing a 40% reduction. We therefore used a regimen of 3,6′-DT treatment at 5 h after CCI in subsequent experiments.

### 2.3. Post-Injury Treatment Reduced TBI-Induced Neurodegeneration

**Figure 3:**
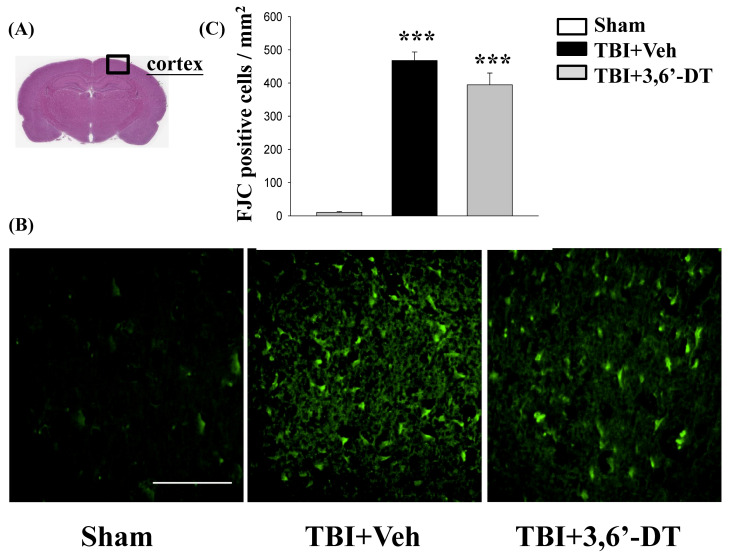
TBI induces neuron degeneration within the contusion regions, and treatment with 3,6′-DT reduced the number of TBI-induced degenerating neurons. (**A**) Representative HE-stained coronal brain section from Sham that shows the area of evaluation. (**B**) Representative photomicrographs showing the presence of fluoro-jade C (FJC)-staining at 24 h in different groups. (**C**) Quantitative comparison of mean densities of FJC-positive cells in the cortical contusion area at 24 h post-injury. Data are presented as mean ± S.E.M. (*n* = 5 in each group). *** *p* < 0.001 compared with the Sham group. Scale bar = 100 µm.

We used fluoro-jade C (FJC), a high affinity fluorescent dye for degenerating neurons, to stain the cortex brain sections from TBI animals with or without treatment. In TBI + Veh animals, FJC-positive degenerating neurons in the cortical contusion margin in the ipsilateral but not in the contralateral hemisphere (Figure 3A), were evident 24 h after CCI (Figure 3B). 3,6′-DT reduced the number of FJC-positive cells from 467.72 ± 25.64/mm^2^ to 394.44 ± 35.64/mm^2^ compared with vehicle treatment (Figure 3C) in these TBI animals.

### 2.4. 3,6′-DT Administered at 5 h Post-Injury Improved Functional Outcomes as Revealed by Behavioral Evaluation at 24 h after CCI

**Figure 4:**
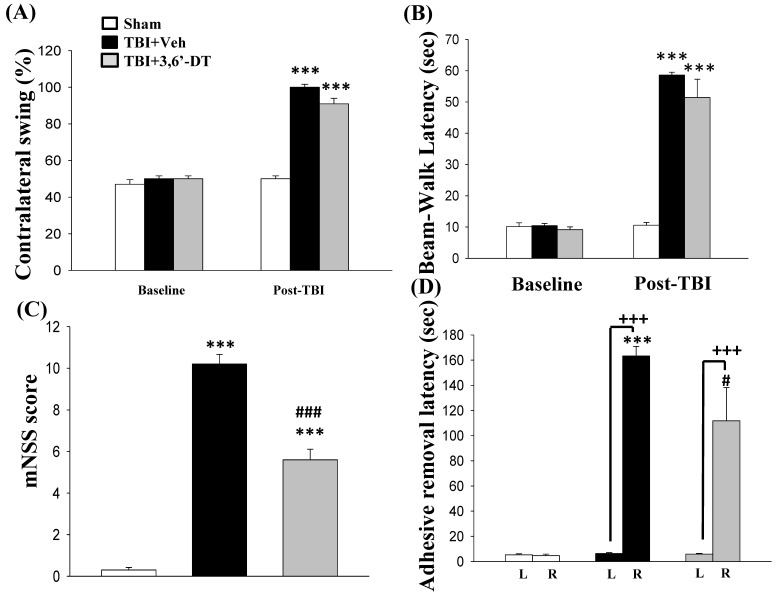
3,6′-DT administered at 5 h post-injury improved functional outcomes as revealed by behavioral evaluation at 24 h after TBI. (**A**) Motor asymmetry measured by the elevated body swing test (EBST). (**B**) Beam walk latency before and after TBI challenge. (**C**) Functional deficits measured by the mNSS score, and (**D**) adhesive removal latency from the left (L) and right (R) forepaw. Data are presented as mean ± S.E.M. (*n* = 5 in each group). *** *p* < 0.001 compared with Sham group; ^#^ *p* < 0.05, ^###^ *p* < 0.001 versus TBI + Veh group; ^+++^ *p* < 0.001 compared with the value of left side.

Motor asymmetry, which was measured by the elevated body swing test (EBST) at 24 h after CCI (Figure 4A), was noted with an increase in contralateral swing ratio in TBI + Veh rats. Specifically, the 3,6′-DT treated group demonstrated a reduced contralateral swing ratios from 100.00 ± 1.58% to 90.90 ± 3.07%.

Likewise, motor coordination impairment in beam walking performance after CCI was observed on the first day in TBI animals (Figure 4B), and 3,6′-DT treated animals showed significantly better performance in beam walking (51.41 ± 5.85 s, *p* < 0.001) than TBI + Veh treated rats (58.59 ± 1.93 s) after injury.

CCI led to severe neurological functional deficits, as measured by mNSS scores (Figure 4C), with TBI + Veh animals showing a significant increase in mNSS scores. Post-injury administration of 3,6′-DT at 5 h ameliorated neurological deficits as revealed by a significantly lower mNSS increment (*p* < 0.001).

Unilateral contusion caused a delay in time for adhesive-removal which was measured by a tactile test at 24 h after injury (Figure 4D). The TBI + Veh group showed functional deficits caused by CCI, and the time required for removing stickers on forelegs was longer than the sham group (163.26 ± 7.69 s and 4.67 ± 1.17 s, respectively. *p* < 0.001). The 3,6′-DT treated TBI group showed a reduced adhesive removal time, as compared with the TBI + Veh group. Taken together, there is a differential effect of 3,6′-DT on somatosensory function, fine motor coordination and other neuronal functions. Our results show that 3,6′-DT improves somatosensory (as revealed by adhesive removal test) and neurological functions (as revealed by mNSS score), but not contralateral swing or beam walking.

### 2.5. Treatment with 3,6′-DT Reduced Apoptotic Neurons in the Cortical Contusion Regions after TBI

**Figure 5:**
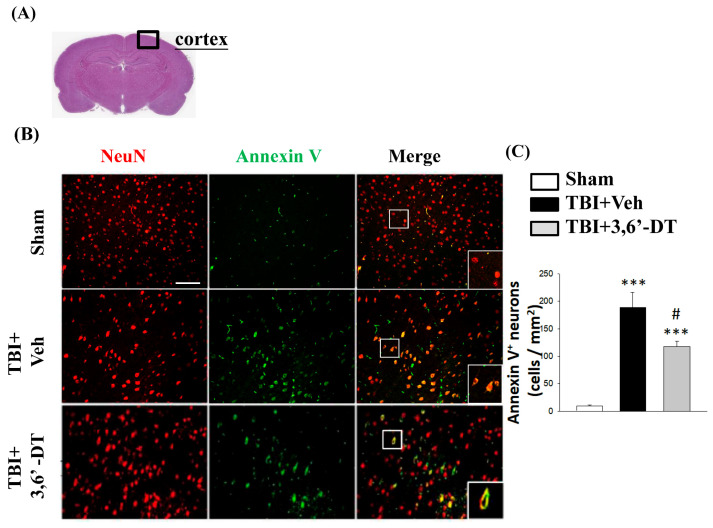
Post-injury administration of 3,6′-DT at 5 h after TBI significantly decreased apoptotic neurons in the cortical contusion regions at 24 h. (**A**) The representative HE-stained coronal section showing the area as indicated by the black square box to compare the fluorescent signals across groups of rats. (**B**) The immunofluorescence of Annexin V and NeuN in cortical brain tissue. The Annexin V immunoreactivity is shown in green, and NeuN (a marker for neurons) is shown in red. The yellow color indicates colocalization. (**C**) The number of Annexin V/NeuN positive cells was elevated when evaluated at 24 h after TBI. Treatment with 3,6′-DT significantly decreased the number of apoptotic neurons compared with TBI + Veh animals. Data are presented as mean ± S.E.M. (*n* = 5 in each group). *** *p* < 0.001 compared with the Sham group. ^#^ *p* < 0.05 compared with the TBI + Veh group. Scale bar = 100 μm.

Since 3,6′-DT treatment significantly improved neurological outcomes, we investigated whether this treatment reduced apoptotic processes in neurons. Cellular changes observed during apoptosis include externalization of phosphatidylserine (PS), a membrane lipid. Translocation of PS to the outer membrane is a marker of early apoptotic processes. We labeled outer membrane levels of PS on the exterior of cells using antibodies specific to Annexin V. This method of detecting early apoptotic cells has been widely used for in vitro and in vivo imaging of apoptosis [32,33]. Specifically, we used immunofluorescence staining to observe neuronal apoptosis at 8 and 24 h after TBI by evaluating a sample area located in the lesioned hemisphere of the brain cortex (Figure 5A). We also used an antibody specific for neuronal nuclei (NeuN) to label neurons. NeuN antibody-labeled neurons are shown with red fluorescence and annexin V antibody-labeled membranes shown with green fluorescence (Figure 5B). The number of Annexin V/NeuN positive cells was determined (yellow fluorescence) in the Sham group (13 ± 1), TBI + Veh group (184 ± 30), and TBI + 3,6′-DT group (117 ± 10) cells/mm^2^ (Figure 5C). There was a significant reduction of colocalized (yellow) neurons in the 3,6′-DT CCI group as compared to CCI vehicle treated animals. In samples evaluated 8 h after CCI, although the numbers of colocalized apoptotic neurons were significantly greater than in shams, there was no significant effect of 3,6′-DT vs. vehicle.

### 2.6. Treatment with 3,6′-DT Suppressed Microglia Activation

**Figure 6:**
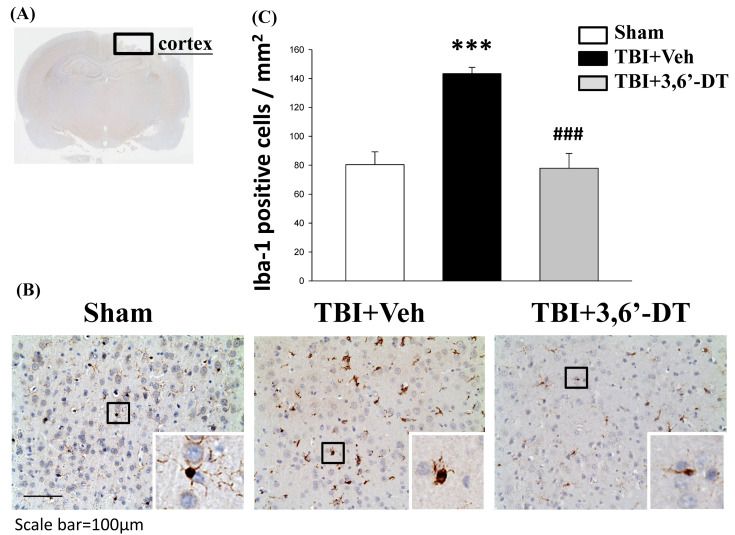
TBI induces microglia activation within the contusion regions, and treatment with 3,6′-DT significantly suppressed microglia activation. (**A**) A low power image of brain section immune-histochemically stained with Iba-1 antibody in cortical regions from a TBI animal showing the contusion site and area of observation (Black square). (**B**) Representative photomicrographs showing the Iba-1-positive cells with amoeboid (activated) or ramified (resting) phenotypes in cortical regions from various animal groups at 24 h after TBI. (**C**) Quantitative comparison of mean densities of Iba-1-positive cells in the cortical contusion area at 24 h post-injury. Microglial activation after TBI, as observed by the increased number and change of their morphologic phenotypes (from ramified to amoeboid), was reduced by 3,6′-DT treatment. Mean ± S.E.M. (*n* = 5 in each group). *** *p* < 0.001 compared with the Sham group. ^###^ *p* < 0.001 compared with the TBI + Veh group.

Microglia were identified by immunohistochemical staining with an antibody against ionized calcium binding adaptor molecule-1 (Iba-1) which is a microglia/macrophage-specific calcium-binding protein. Microglial activation can be revealed by the increased number as well as change of their morphologic phenotypes (from ramified to amoeboid). As illustrated in Figure 6, the Iba-1-positive cells with amoeboid (actived) or ramified (resting) phenotypes were visulized in cortical regions from various animal groups (Figure 6A,B). Compared to the Sham group, the TBI + Veh group displayed fewer Iba-1-positive cells with the ramified (resting) phenotype but more cells with the amoeboid (activated) phenotype in the injured cortex. However, compared to the TBI + Veh group, the TBI + 3,6′-DT group displayed increased numbers of cells with the ramified phenotype and fewer cells with the hypertrophic or amoeboid phenotype in the ipsilateral cortex (Figure 6C), indicating 3,6′-DT suppressed microglia activation.

### 2.7. 3,6′-DT Administered at 5 h Post-Injury Downregulates Injury-Induced Cytokine mRNA Expression at 8 h after CCI

**Figure 7:**
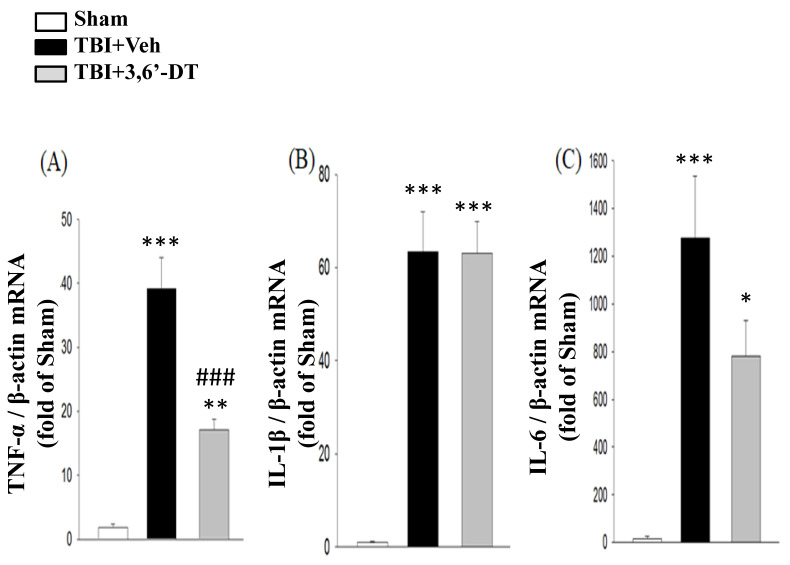
Treatment with 3,6′-DT at 5 h after TBI reduced injury-induced mRNA expression of cytokines at 8 h after TBI in the ipsilateral hemisphere cortex. TBI-induced elevations in mRNA levels of (**A**) *TNF-α*, (**B**) *IL-1β* and (**C**) *IL-6*. Data are presented as mean ± S.E.M. (*n* = 5 in each group). * *p* < 0.05, ** *p* < 0.01, *** *p* < 0.001 compared with the Sham group. ^###^ *p* < 0.001, compared with the TBI + Veh group.

To evaluate the expression of *TNF-α* mRNA, reverse transcription-qPCR was used to measured gene transcription at 8 h after CCI in the cortical contusion regions (Figure 7A–C). The results revealed that following injury, *TNF-α* gene expression was markedly elevated in the vehicle treated group compared with the corresponding regions of the sham group (39.17 ± 4.89-fold, *p* < 0.001). We also evaluated pro-inflammatory cytokines, *IL-1β* and *IL-6* gene expressions and found that *IL-1β* and *IL-6* mRNA levels in the cortex were significantly increased at 8 h after CCI (63.38 ± 8.60-fold and 1275.35 ± 261.70-fold, *p* < 0001). Treatment with 3,6′-DT at 5 h significantly decreased *TNF-α* mRNA levels 8 h after injury, compared to the TBI + Veh group; yet, no significant differences in *IL-1β* and *IL-6* levels were observed.

### 2.8. 3,6′-DT Reduced Pro-Inflammatory Cytokine Protein Expression at 8 h after TBI in the Ipsilateral Hemisphere

**Figure 8:**
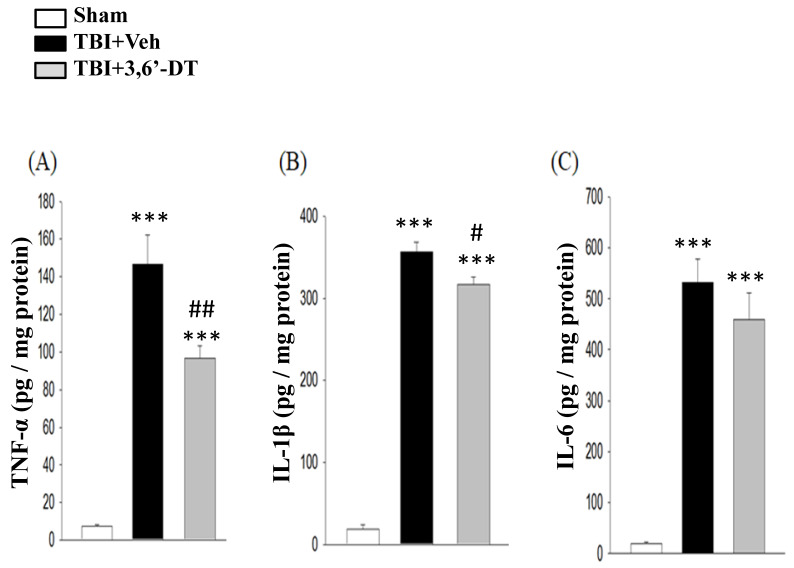
Post-injury administration of 3,6′-DT at 5 h after TBI significantly reduced the elevated tissue levels of inflammatory cytokine proteins measured at 8 h. (**A**) TNF-α, (**B**) IL-1β, and (**C**) IL-6 protein levels in TBI + Veh rats were significantly elevated in the ipsilateral cortex at 8 h after injury. Administration of 3,6′-DT mitigated the rise in these three cytokine levels, which reached statistical significance for TNF-α, and IL-1β, but not IL-6. Data are presented as mean ± S.E.M. (*n* = 5 in each group). *** *p* < 0.001 compared with the Sham group. ^#^ *p* < 0.05, ^##^ *p* < 0.01, ^###^ *p* < 0.001 compared with the TBI + Veh group.

To examine whether post-treatment with 3,6′-DT at 5 h reduces the tissue levels of pro-inflammatory cytokine protein concentrations at 8 h after CCI, we compared TNF-α, IL-1β and IL-6 levels in sham, TBI + Veh treated, and TBI + 3,6′-DT treated groups by ELISA (Figure 8A–C). Basal protein levels of these pro-inflammatory cytokine were low in the cortical tissue of the sham group. Following CCI, protein levels of these pro-inflammatory cytokines in the ipsilateral cortex were significantly elevated at 8 h in the vehicle treated TBI group (*p* < 0.001); TNF-α, 146.46 ± 15.65 pg/mg, IL-1β, 356.51 ± 12.04 pg/mg, IL-6, 531.38 ± 46.48 pg/mg. Post-injury treatment with 3,6′-DT reduced the CCI-induced increase in cortex levels of these three pro-inflammatory cytokines.

### 2.9. Treatment with 3,6′-DT Reduces CCI-Induced Oxidative Damage in the Cortical Contusion Regions after CCI

**Figure 9:**
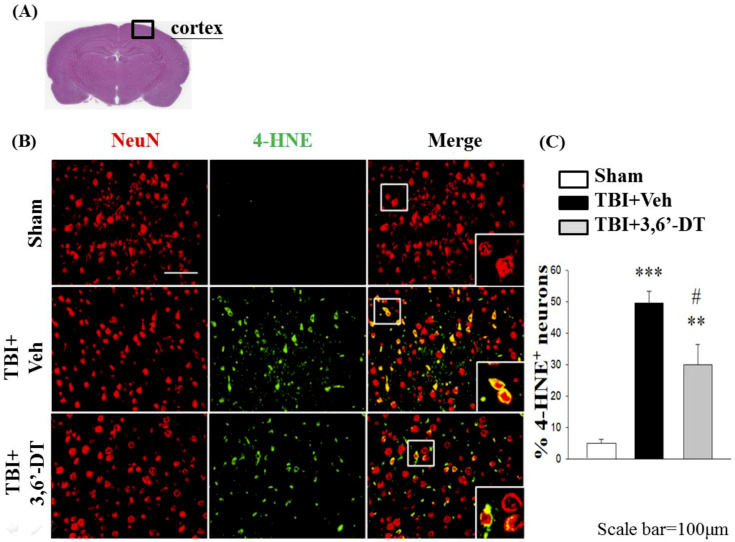
Administration of 3,6′-DT at 5 h post-injury reduced injury-induced lipid peroxidation product in the cortical contusion regions at 24 h. (**A**) The representative HE-stained coronal section showing the area as indicated by the black square box to compare the fluorescent signals across 3 groups of rats. (**B**) The immunofluorescence of 4-hydroxynonenal, 4-HNE (lipid peroxidation product), and NeuN in cortical brain tissue. The 4-HNE immunoreactivity is shown in green, and NeuN (a marker for neurons) is shown in red. The yellow cells indicate colocalization. (**C**) There was a significant CCI TBI-induced elevation in the number of 4-HNE positive neurons that was significantly ameliorated in the TBI + 3,6′-DT group. Data are presented as mean ± S.E.M. (*n* = 5 in each group). ** *p* < 0.01, *** *p* < 0.001 compared with the Sham group. ^#^ *p* < 0.05 compared with the TBI + Veh group. Scale bar = 100 µm.

**Figure 10:**
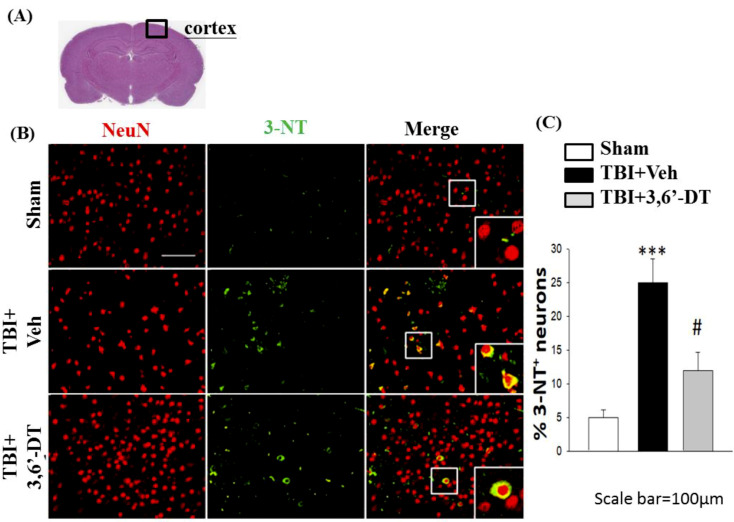
Administration of 3,6′-DT at 5 h post-injury reduced injury-induced tyrosine nitration product in the cortical contusion regions at 24 h. (**A**) The representative HE-stained coronal section indicates the area used to compare the fluorescent cell observations in different animal treatment groups. (**B**) The immunofluorescence of 3-nitrotyrosine, 3-NT (tyrosine nitration product mediated by reactive nitrogen species), and NeuN in cortical brain tissue. The 3-NT immunoreactivity is shown in green, and NeuN is shown in red. The yellow color indicates colocalization. (**B**) Compared to TBI + Veh animals there was a significant decrease in the number of 3-NT/NeuN positive cells in the TBI + 3,6′-DT group. Data are presented as mean ± S.E.M. (*n* = 5 in each group). *** *p* < 0.001 compared with the Sham group. ^#^ *p* < 0.05 compared with the TBI + Veh group. Scale bar = 100 µm.

The generation of oxidative stress and reactive oxygen species are shown using immunofluorescence of 4-hydroxynonenal (4-HNE, Figure 9) and 3-nitrotyrosine (3-NT, Figure 10) at 24 h after CCI. With both markers, the reactive oxygen species are shown in green and NeuN, the neural marker, is shown in red. Colocalization in neurons is shown in yellow. Staining for both 4-HNE (Figure 9) and 3-NT (Figure 10) indicated that there was a significant increase in the positive, dual staining of cells in TBI + Veh vs. sham group, and that this increase was significantly ameliorated by treatment with 3,6′-DT when administered 5 h after CCI.

## 3. Discussion

Our data demonstrate the utility of a post-injury, single administration of a novel experimental drug, 3,6′-dithiothaliomide, in a rat model of moderate TBI. Our findings illustrate significant drug-induced reductions in cortical tissue loss, improvements in a range of functional outcomes associated with attenuations in injury-induced microglia activation elevations of pro-inflammatory cytokine mRNA and protein, and decreased numbers of degenerating and apoptotic neurons. Thus, we have documented that early post-injury administration of 3,6′-DT is able to reverse functional and histological defects 24 h following a moderate TBI. The secondary pathological processes in brain after TBI are not singular events, but occur over different time courses. Thus, for example, some of the molecular changes involving apoptosis and neuroinflammation are maximal at 8 hrs after injury. In contrast, behavioral changes are best studied after 24 h to avoid confounds of the anesthesia given during the TBI surgery. Cell loss and contusion size are also best studied after 24 h when the extent of injury has stabilized. To maximize the “signal to noise” ratio for these molecular and cellular/behavioral changes and the effects of experimental treatments, different time courses of analysis were selected based on our previous studies. Since injury-induced inflammatory responses that contribute to the histological and behavioral pathophysiology of TBI were predominantly affected, our findings suggest that 3,6′-DT has neuroprotective properties, and that functional improvement is perhaps provided, in large part, through the modulation of inflammation.

The differentiation between “mild” and “moderate” TBI derives primarily from clinical nomenclature using the Glasgow Coma Scale (GCS). Levels of injury in the 13–15 range are considered mild, and there is usually no evidence of a loss of brain tissue on CAT or MRI scans. Moderate injuries are often associated with brain scan changes such as brain tissue loss, white matter injuries, etc. Although not perfect, these definitions are often translated to preclinical studies focusing on whether there are overt and significant histological and anatomical changes in brain associated with behavioral and other parameters. Neurological and functional deficits were both evident and readily quantifiable in the experimental TBI animal model used in the current study, and are likewise common neurological sequelae in patients with brain injury. Since TBI is associated with complex changes in functional outcome, it is essential to evaluate a combination of behavioral evaluations in order to assess functional deficits following TBI, especially measurements of sensorimotor dysfunction, motor asymmetry and impairment of motor coordination order to correlate functional and histological outcomes [34]. In this regard, we found that 3,6′-DT significantly improved functional deficits which correlated well with the histological findings estimated by Nissl (HE) and FJC staining. Post-TBI treatment with 3,6′-DT showed smaller contusion volumes and fewer degenerating neurons compared with the vehicle-treated group, resulting in an amelioration of trauma-induced neuronal loss and improvement of neurobehavioral outcomes.

A rapid upsurge in the inflammatory response preceded neuronal cell death which was strongly correlated with the increased secondary injury after TBI, manifested by an expansion of contusion volume. Although the inflammatory response is part of the innate immune system and is key to initiating reparative mechanisms, its over activation can contribute to, and drive degenerative cascades [35,36]. Attenuation of the inflammatory response to reduce tissue damage following TBI is therefore a reasonable therapeutic strategy [37]. In the current study, we demonstrated that post-injury administration with 3,6′-DT reduced pro-inflammatory cytokine mRNA expression and protein levels in ipsilateral hemisphere cortex in parallel with reduced cortical contusion volume and neurological deficits. There is substantial evidence that pro-inflammatory cytokines, TNF-α, IL-1β and IL-6 can operate at very low concentrations on many cell types in the CNS or periphery, and can contribute to neurodegeneration in acute brain injury [38,39]. Blocking cytokine receptors or inhibiting pro-inflammatory cytokine activation therefore has major neuroprotective potential [8,40,41]. In addition, TNF-α is an inducer and enhancer of inflammatory reactions via the activation of glia and blood cells, such as macrophages and neutrophils. Depending on the signaling pathway, TNF-α can make trauma and oxidative stress more deleterious in the brain. TNF-α is regulated both transcriptionally and post-transcriptionally, the latter via elements within its 3′-untranslated region that repress or augment translation. Excessive or uncontrolled upregulation can also enhance glutamate release from astrocytes that can lead to glutamate excitotoxicity and blood-brain barrier dysfunction [36,42] via a self-propagating pathological cascade of neuroinflammation and neuronal loss [7], further contributing to the production of multiple downstream cytokines, like IL-6, IL-1β and TNF-α itself [43]. Our findings suggest that TBI is associated with a significant rise in TNF-α protein levels at 8 hr, which is in accordance with other preclinical studies [8,30,40,44]. This rise in TNF-α protein levels was substantially downregulated with 3,6′-DT treatment, demonstrating that its cellular actions found in RAW cells translate into in vivo actions in brain. Elevated brain protein levels of TNF-α, IL-1β and IL-6 have also been detected in human CSF and serum after brain injuries [41,45,46]. Finally, an increase of cytokines is also associated with a wide range of acute and chronic neurodegenerative insults including stroke and AD, in addition to TBI, and likewise, these time-dependent alterations appear to drive neuronal dysfunction and loss [26,29,47].

Although the elevation of TNF-α levels in the early hours after TBI may be harmful, cytokine balance is likely essential for long-term recovery from injury. The overproduction of pro-inflammatory cytokines in the early stages of injury appears to be detrimental to brain tissue, whereas low levels and a regulated balance of these cytokines during the recovery stage could improve outcomes for injured tissues and enhance repair processes [11,48]. One option to attenuate TNF-α is to use an antibody approach to inhibit and clear TNF-α before it could reach its target [44,49,50]; this has effectively been achieved in the treatment of rheumatoid arthritis. However, brain uptake of antibodies is limited unless there is substantial disruption of the blood-brain barrier or it is administered perispinally [51], which limits the potential utility of this approach. Our aim was to use a small molecular weight TNF-α synthesis inhibitor that can readily access the brain to maintain lowered TNF-α protein levels after TBI [23]. The pathophysiology of TBI is amplified by secondary injuries that include oxidative damage, cell necrosis, hypoxia, ischemia, brain edema and impaired function [52]. These processes over time can lead to early dementia onset, Alzheimer’s disease and Parkinson’s disease, as well as to other chronic neurodegenerative disorders [53,54,55].

Taken together, 3,6′-DT might represent a novel potential therapy for TBI [27,28,29,30]. Our dose of 3,6′-DT (28 mg/kg, equipotent to 25 mg/kg thalidomide) compares favorably with those of thalidomide used in humans, where doses of up to 1200 mg are administered. This view is further validated by prior studies demonstrating the ability of 3,6′-DT to reduce neuroinflammation in rodent models of stroke, mild TBI and Alzheimer’s disease that likewise resulted in neuroprotective actions and the attenuation of functional deficits.

## 4. Materials and Methods

### 4.1. Cellular Studies

RAW 264.7 cells, purchased from ATCC (Manassas, VA, USA), were grown in DMEM media supplemented with 10% FCS, penicillin 100 U/mL and streptomycin 100 g/mL. The cells were incubated at 37 °C and 5% CO_2_, and were propagated as described in ATCC guidelines. RAW 264.7 cells (~400 × 10^3^) were seeded in 24 well plates and allowed to equilibrate overnight. The following day, the seeding media was aspirated and replaced with 1 mL of fresh media. After 2 h, 3,6′-DT dissolved in 100 % tissue culture grade dimethylsulphoxide (DMSO, Sigma Aldrich, Saint Louis, MO, USA) was added to the wells. 3,6′-DT was synthesized to a chemical purity of >99.5%, and chemical characterization was undertaken by a combination of 1H NMR, 13C NMR and GC/MS analyses (Bruker AC-300 spectrometer, Billerica, MA, USA) [23], together with elemental analyses (Atlantic Microlab, Inc., Norcross, GA, USA). The drug concentrations to which the cells were exposed were 1, 10, 30 and 60 µM 3,6‘-DT (*n* = 3–4 for each group).

One hour after the addition of 3,6′-DT or DMSO vehicle control, the cells were challenged with lipopolysaccharide (LPS, SIGMA, serotype 055:B5, at a concentration of 30 ng/mL). Twenty to 24 h after the addition of LPS, the media was collected for the analysis of TNF-α concentrations (Mouse TNF-α ELISA MAX™ Deluxe Cat # 430906), nitrite concentrations, (Griess Reagent System, PROMEGA, Cat # G2930, Madison, WI, USA) and lactate dehydrogenase activity in the media as evidence of drug-induced cellular toxicity (In Vitro Toxicology Assay Kit, Lactic Dehydrogenase based, SIGMA, Cat # Tox7-1KT).

Cells were subsequently harvested and used for protein blotting studies to detect inducible nitric oxide synthase (iNOS (D6B6S), Rabbit mAb #13120, Cell Signaling Technology) and α-tubulin (Monoclonal Anti-α-Tubulin antibody, Cat # T6074, SIGMA). Conventional Western Blotting methods were employed. In brief, 15 µg of total RAW cell protein was separated in Bis-Tris gels (Thermo Fisher Scientific, NuPAGE™ 4–12%, Cat # NP0336BOX, Waltham, MA, USA); after electrophoresis the proteins were transferred to polyvinylidene difluoride (PVDF) membrane (Thermo Fisher Scientific, Cat # 88520). The membrane was then blocked with a 5% milk protein in Tris Buffered Saline with Tween (0.1%) solution (TBST) for 1 h at room temperature, after which primary antibodies were incubated over night at 4 °C. The next day, the membrane was washed and then incubated with an appropriate antibody conjugated with horseradish peroxidase (HRP) for 1 h in TBST at room temperature. After a series of washes, the membrane was exposed to SuperSignal™ West Pico PLUS Chemiluminescent Substrate (Thermo Fisher Scientific, Cat # 34580) and protein levels were assessed on Amersham^®^ Hyperfilm^®^ ECL™ (GE Healthcare, Cat # 95017-673, Chicago, IL, USA). Band densitometry was performed using Image J (NIH); iNOS band density normalized to α-tubulin band densities.

### 4.2. In Vivo Studies

All animals were treated according to the International Guidelines for animal research, and the animal protocol used in this study was reviewed and approved by the Institutional Animal Care and Use Committee or Panel (IACUC/IACUP) committee of Taipei Medical University (Approval number LAC-2015-0051, 01-April, 2015). Male Sprague-Dawley (SD) rats (250–300 g, body weight) were housed in groups in a temperature (21~25 °C) and humidity (45~50%) controlled room with a 12-h light/dark cycle and ad libitum access to pellet chow and water. We used an animal model of TBI using controlled cortical impact (CCI) as described previously [56,57,58,59], with control of body temperature during and after CCI surgery.

The CCI injury procedure was performed as described previously [56,57,58,59]. Totally 60 rats were randomly placed into 3 groups (*n* = 5 in each group, sacrificed 8 h and 24 h after TBI to sum up as 30 animals perfused with paraformaldehyde for tissue sectioning and staining and another 30 rats for biochemical measurements). (i) Sham injury, (ii) TBI + Vehicle and (iii) TBI+3.6′ DT. A CCI instrument with a rounded metal tip (5 mm diameter), an impact velocity of 4 m/s and a deformation depth of 2 mm below the dura was used. Body temperature was monitored throughout surgery by using a rectal probe; the temperature was maintained at 37.0 ± 0.5 °C using a heated pad during recovery from anesthesia. Thereafter, animals were euthanized at 8 or 24 h after TBI and their brains were processed for the following experiments to examine the neuroprotective effects of 3.6′-DT. These analysis times are in accord with peak levels of apoptosis, as well as behavioral and imaging.

### 4.3. Drug Administration

3,6’-Dithiothalidomide (3,6′-DT) was freshly prepared prior to each study. In these animal studies, 3,6’-dithiothalidomide was prepared as a suspension in 1% carboxymethyl cellulose to provide a final concentration of 28 mg/kg (0.1 mL/100 g) body weight for injection in rats and administered by the intraperitoneal (i.p.) route. This concentration of 3,6’-DT is equimolar to 25 mg/kg of thalidomide. Vehicle (Veh)-treated rats received 1% carboxymethyl cellulose (i.p.). All rats were euthanized for histological and biological analysis at 8 or 24 h after TBI.

### 4.4. Behavioral Evaluation of Neurological Outcomes

Behavioral testing was performed before TBI and at 24  h thereafter. The evaluation consisted of an elevated body swing test (EBST), a modified neurological severity score (mNSS) assessment, and a beam walk test. These procedures were performed as previously described [56,57,59]. In addition, a tactile removal test was performed to appraise somatosensory function (paw dexterity) by measuring the ability of the animal to undertake sensitive paw to mouth movements. Specifically, two small adhesive stickers were placed on the distal-radial region of the wrist of each rat’s forelimb, and the time (up to 3 min) to remove these was recorded. Rats were pre-trained daily for 3 days before TBI, and evaluated (for five trials) 24 h after TBI. The mean time taken to remove the stickers from the last trial was used to generate a latency time for sticker removal from each paw [59]. Finally, fine motor coordination was evaluated by the use of the beam walk test [60], as described previously [59]. Notably, each of these paradigms was performed by an observer blinded to the experimental groups.

### 4.5. Contusion Volume

To measure the volume of TBI-induced injury in the ipsilateral cortex 24 h after TBI, cresyl violet-stained sections were digitized and analyzed using Image J (National Institutes of Health, Bethesda, MD, USA). The volume was computed by adding the injury areas and multiplying by the inter-slice distance (500 µm). Hemispheric tissue loss was expressed as a percentage which was calculated by the use of the following formulae: [(contralateral hemispheric volume − ipsilateral hemispheric volume)/(contralateral hemispheric volume)×100%], as previously reported [61]. We used the protocol as previously described [56,57,59].

### 4.6. Fluoro-Jade C (FJC) Staining

FJC, a derivative of polyanionic fluorescein, selectively binds to degenerating neurons. Using a FJC ready-to-dilute staining kit (TR-100-FJ, Biosensis, UK), we identified degenerating neuronal cells in cortex tissues according to the manufacturer’s protocol with some modifications [57,59]. Brain sections from the different treatment groups were deparaffinized, rehydrated, incubated in distilled water for 3 min, and then incubated in a solution of potassium permanganate (1:15) for 10 min. Next, they were rinsed in distilled water for 2 min, and then incubated in the FJC solution (1:25) for 15 min. After incubation, slides were washed and mounted on coverslips with Vecta-shield mounting medium (Vector Laboratories, Burlingame, CA, USA). All sections were observed and photographed using a fluorescent inverted microscope (IX70, Olympus, Japan). Numbers of FJC-positive cells were counted in three randomly selected fields per slide by means of SPOT image analysis software (Diagnostic Instruments, Sterling Heights, MI, USA). Numbers of FJC-positive cells observed on the slides from different treatment groups were counted and used to generate a mean number per treatment group.

### 4.7. RNA Extraction, Reverse Transcription, and Real-Time Quantitative PCR (qPCR)

Total RNA was extracted by using TRIzol reagent (Life Technologies, Carlsbad, CA, USA). The purity and quality of RNA were confirmed by defining the ratio of absorbance at 260 and 280 nm wavelengths (NanoDrop^®^ ND-1000, Thermo Scientific, Waltham, MA, USA). A sample of 3 µg total RNA was reverse transcribed into cDNA. For mRNA measurement, diluted cDNA was amplified using the Rotor-Gene SYBR Green PCR Kit (Qiagen, Hilden, Germany) in a Rotor-Gene Q 2plex HRM Platform (Qiagen). Reaction conditions were carried out for 35–40 cycles (5 min at 95 °C, 5 s at 95 °C and 10 s at 60 °C). All procedures for RNA extraction and qPCR have been described previously [57,58,59].

The primers were designed using reported cDNA sequences: **1.** *TNF-α*, Forward 5′-CTC TTC TCA TTC CCG CTC GTG-3′ and Reverse 5′-GGA ACT TCT CCT CCT TGT TGG G-3′; **2.** *IL-1β*, Forward 5′-GTT TGA GTC TGC ACA GTT CCC-3′ and Reverse 5′-CAA CTA TGT CCC GAC CAT TGC-3′; **3.** *IL-6*, Forward 5′-TTC TTG GGA CTG ATG TTG TTG AC-3′ and Reverse 5′-AAT TAA GCC TCC GAC TTG TGA AG-3′; **4.** β-actin, Forward 5′-GAC CCA GAT CAT GTT TGA GAC CTT C-3′and Reverse 5′-GAG TCC ATC ACA ATG CCW GTG G-3′.

### 4.8. Enzyme-Linked Immunosorbent Assay (ELISA)

Rat cortical tissues were harvested 8 h post-injury (*n* = 5), homogenized in lysis buffer (RIPA, Sigma, St Louis, MO), and centrifuged at 8000× *g* for 10 min at 4 °C. The resulting supernatant was collected and stored at −80 °C until the time of analysis of protein levels by specific ELISAs for rat TNF-α, IL-1β, and IL-6 (R&D System (RTA00; RLB00; R6000B). The sample protein content was determined by the use of the BCA assay (Pierce™ BCA Protein Assay Kit, #23225, Waltham, MA, USA), and levels of cortical protein were determined by following the manufacturers protocol. Cortical cytokine protein levels were normalized to a pg/mg of tissue unit.

### 4.9. Immunohistochemistry (IHC) or Double Immunofluorescence

Brain sections from sham, TBI + Veh rats, and TBI rats treated with 3,6’-DT were deparaffinized and rehydrated. Serial sections (10 µm) through the cerebral cortex were stained with hematoxylin and eosin for microscopic evaluation. We then used primary antibodies against of Iba-1 protein (GeneTex, GTX100064, 1:300) to identify microglia, NeuN (Cell Signaling, #12943, 1:200) to identify neurons, and Annexin V (Santa Cruz, Sc-74438; 1:500, Island, CA, USA) to identify apoptotic cells in brain sections from various groups of animals. Cellular oxidative stress was detected by primary antibody against the lipid peroxidation product 4-hydroxunonenal (4-HNE, Abcam, ab46545, 1:250, Cambridge, England) and protein nitration product 3-nitrotyosine (BioVision, 5412-100, 1:250, Lausen, Switzerland) in the ipsilateral cortex of rats. For Immunohistochemistry, after incubation at 4 °C overnight, the sections were washed and incubated with VECTASTAIN Elite ABC HRP Kit (Peroxidase) at room temperature for 1hr. For double immunofluorescence, after incubation at 4 °C overnight, we washed the sections and incubated with: Alexa 594 (Jackson ImmunoResearch Lab., 115-585-062, 1:200, Philadelphia, PA, USA) + Alexa 488 (Invitrogen, A11034, 1:200, Carlsbad, CA, USA), Alexa 594 (Jackson, 111-585-003, 1:200) + Alexa 488 (Jackson, 115-545-062, 1:200) at room temperature for 1hr. We then counted the numbers of labeled cells in each section by using SPOT image analysis software (Diagnostic Instruments, Sterling Heights, MI, USA) as described in previous publications [57,58,59].

### 4.10. Statistical Analysis

Data were evaluated between groups with one-way analysis of variance (ANOVA) followed by Tukey or Bonferroni tests (GraphPad InStat Version 3.05, San Diego, CA, USA) when appropriate for multiple comparisons. The mean and standard error of the mean (SEM) were calculated using Sigma Plot and Stat version 2.0 from Jandel Scientific, San Diego, CA. Bar graphs are presented as mean ± SEM values.

## 5. Conclusions

Our results indicate that 3,6′-DT has neuroprotective properties in a moderate CCI model of TBI, whereby it attenuates functional deficits mainly through ameliorations in mediators of inflammation that reduce levels of secondary brain injury. Although this experimental drug has yet to be tested in humans, the greater potency and brain penetrability of 3,6′-DT compared to that of thalidomide, taken together with its good therapeutic window observed in the present TBI study, suggest a possible potential use for thalidomide analogs such as 3,6′-DT in TBI patients.
