# Correction: Batsaikhan et al. Post-Injury Neuroprotective Effects of the Thalidomide Analog 3,6′-Dithiothalidomide on Traumatic Brain Injury. *Int. J. Mol. Sci.* 2019, *20*, 502

**DOI:** 10.3390/ijms27010393

**Published:** 2025-12-30

**Authors:** Buyandelger Batsaikhan, Jing-Ya Wang, Michael T. Scerba, David Tweedie, Nigel H. Greig, Jonathan P. Miller, Barry J. Hoffer, Chih-Tung Lin, Jia-Yi Wang

**Affiliations:** 1 Graduate Institute of Medical Sciences, College of Medicine, Taipei Medical University, 250 Wu-Xing Street, Taipei 11031, Taiwan; buyndlgr@gmail.com (B.B.); gaeajyw911real@hotmail.com.tw (J.-Y.W.); sss880205@gmail.com (C.-T.L.); 2 Drug Design & Development Section, Translational Gerontology Branch, Intramural Research Program, National Institute on Aging, NIH, Baltimore, MD 21224, USA; mike.scerba@nih.gov (M.T.S.); tweedieda@grc.nia.nih.gov (D.T.); greign@grc.nia.nih.gov (N.H.G.); 3 Department of Neurological Surgery, Case Western Reserve University, Cleveland, OH 44106, USA; Jonathan.Miller@uhhospitals.org (J.P.M.); bjh82@case.edu (B.J.H.); 4 Department of Physiology, School of Medicine, College of Medicine, Taipei Medical University, Taipei 11031, Taiwan

## Error in Figure

The authors wish to make the following corrections to this paper [1]:

The authors have realized an unfortunate error in the selected representative image for Sham > and TBI + Veh animals. The corrected Figure 5B appears below.

Corresponding to the corrected figure panel, the authors added statistical values (mean ± SEM) in the statement explaining Figure 5C. A correction has been made to the Section 2.5, the 9th Sentence: The number of Annexin V/NeuN positive cells was determined (yellow fluorescence) in the Sham group (13 ± 1), TBI + Veh group (184 ± 30), and TBI + 3,6′-DT group (117 ± 10) cells/mm^2^ (Figure 5C).

The authors state that the scientific conclusions are unaffected. This correction was approved by the Academic Editor. The original publication has also been updated.

## Figures and Tables

**Figure 5:**
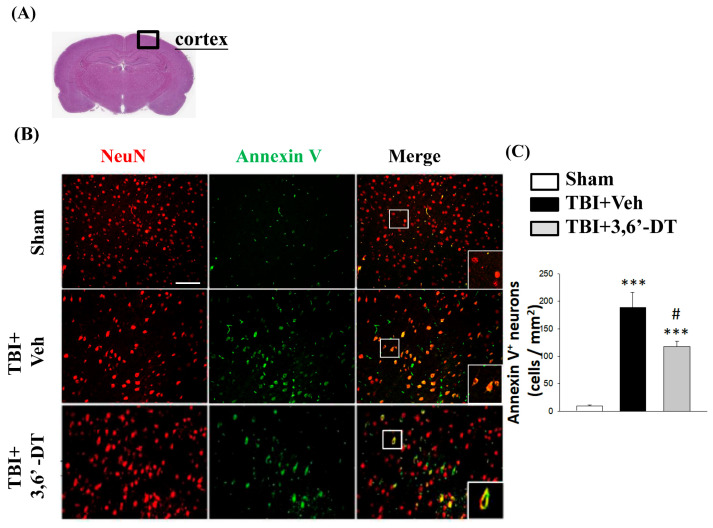
Post-injury administration of 3,6′-DT at 5 h after TBI significantly decreased apoptotic neurons in the cortical contusion regions at 24 h. (**A**) The representative HE-stained coronal section showing the area as indicated by the black square box to compare the fluorescent signals across groups of rats. (**B**) The immunofluorescence of Annexin V and NeuN in cortical brain tissue. The Annexin V immunoreactivity is shown in green, and NeuN (a marker for neurons) is shown in red. The yellow color indicates colocalization. (**C**) The number of Annexin V/NeuN positive cells was elevated when evaluated at 24 h after TBI. Treatment with 3,6′-DT significantly decreased the number of apoptotic neurons compared with TBI + Veh animals. Data are presented as mean ± S.E.M. (*n* = 5 in each group). *** *p* < 0.001 compared with the Sham group. ^#^ *p* < 0.05 compared with the TBI + Veh group. Scale bar = 100 μm.
